# Fabrication and Functional Modification Strategies of Squid Ink-Derived Nanoparticles: From Natural Melanin to Multifunctional Biomaterials

**DOI:** 10.3390/md24030089

**Published:** 2026-02-24

**Authors:** Jung Min Shin

**Affiliations:** Department of Polymer Science and Engineering, Korea National University of Transportation, Chungju 27469, Republic of Korea; jmshin@ut.ac.kr; Tel.: +82-043-841-5424

**Keywords:** squid ink, melanin nanoparticle, fabrication, surface modification, nanomaterials, biofunctionalization

## Abstract

Squid ink has recently garnered considerable attention as a natural melanin source for the development of biocompatible nanomaterials. Although numerous studies have explored the biological and therapeutic applications of squid ink, the fabrication and modification strategies for squid ink-derived nanoparticles (SINPs) have yet to be comprehensively reviewed. This paper provides an integrated overview of current extraction, purification, and functionalization strategies for SINPs, with a particular focus on how functionalization approaches modulate their physicochemical characteristics and biological behaviors. The review begins by outlining the natural mechanisms of melanin formation and summarizing common extraction methods—including centrifugation, ultrasonication, and dialysis. Subsequently, various surface modification and hybridization techniques—including polymer coating, incorporation of metallic elements (e.g., Se and Fe), and loading of photosensitizers—are compared in terms of their contributions to functional enhancement. Finally, the challenges of reproducibility, batch-to-batch variability, and scalable manufacturing are discussed, outlining future directions for the development of squid ink-derived nanomaterials into standardized biomedical platforms.

## 1. Introduction

Marine organisms have long served as a valuable source of bioactive compounds across diverse fields, including biotechnology, biomedical engineering, and medicine [1,2,3]. Marine-derived materials, distinguished by their exceptional biocompatibility, unique physicochemical properties, and ease of modification, are increasingly utilized in disease treatment and transplantation, drawing significant research interest [4]. For example, polysaccharides extracted from marine algae function as hydrophilic components in polymeric nanoparticles and can be chemically conjugated with therapeutic agents to form polymer–drug conjugates, which have been widely applied in nanomedicine, including chemotherapy, radiotherapy, and immunotherapy [5]. Unlike conventional small-molecule chemotherapeutics, nanomedicines offer enhanced drug retention and penetration within tumor tissues through passive targeting mediated by the enhanced permeability and retention (EPR) effect [6]. In addition, nanomedicines equipped with active targeting ligands can directly recognize and bind specific molecular targets, thereby maximizing therapeutic efficacy while reducing off-target toxicity.

Melanin is a natural pigment broadly distributed among living organisms, where it plays vital roles in photoprotection, free radical scavenging, and metal-ion chelation [7]. Structurally, it is a heterogeneous indole-based biopolymer whose physicochemical properties vary depending on its subtype, such as eumelanin or pheomelanin [8]. Owing to its intrinsic biocompatibility, photothermal conversion capability, antioxidative activity, and broadband light absorption, melanin has gained increasing attention as a versatile biomaterial for diverse biomedical and nanotechnological applications, including drug delivery, imaging, phototherapy, and tissue regeneration [9,10,11].

Despite the burgeoning interest in melanin-based platforms, the advancement of melanin-derived nanomaterials has been significantly impeded by several inherent drawbacks of synthetic analogs. Specifically, melanins often exhibit pronounced structural heterogeneity and suboptimal intrinsic bioactivity and involve labor-intensive fabrication processes that necessitate harsh chemical conditions [12]. Consequently, there is an intensifying demand for natural melanin sources, which offer superior biocompatibility, enhanced safety profiles, and sustainable production pathways. Natural melanin possesses unique molecular architectures and inherent biological functionalities that are challenging to replicate via chemical synthesis, thereby positioning it as a highly promising candidate for the development of next-generation functional nanomaterials.

Among various natural sources, squid ink represents a prolific reservoir of melanin and integrated bioactive constituents, including polysaccharides, amino acids, proteins, and lipids [13]. This unique biochemical composition underpins its multifaceted physiological activities, distinguishing it from isolated synthetic variants. Over the past few years, squid ink-derived melanin and its nano-engineered formulations have gained considerable attention due to their exceptional combination of biocompatibility, intrinsic therapeutic properties, and facile extractability [14,15]. The growing recognition of squid ink as a versatile biomaterial is further supported by its demonstrated potential in cancer therapy, antimicrobial interventions, and immunomodulation. By leveraging these inherent properties, squid ink-based nanostructures offer a compelling paradigm for the design of multifunctional nanoplatforms in advanced biomedical engineering.

Accordingly, squid ink-derived nanoparticles (SINPs) have been extensively investigated for a diverse array of biomedical applications, driven by their superior biocompatibility, low immunogenicity, and innate therapeutic functionalities [16,17]. A growing body of literature underscores the anticancer, anti-inflammatory, and immunomodulatory properties of SINPs, facilitating their deployment as potent multifunctional agents. Specifically, the high melanin content within squid ink confers superior photothermal and photodynamic capabilities, which have been integrated into PTT and PDT platforms to enhance cancer treatment efficacy [16]. Beyond such oncological applications, these nanomaterials have demonstrated significant promise in wound healing and tissue regeneration, largely attributed to their robust ROS-scavenging capacity and ability to modulate cellular microenvironments.

Despite this burgeoning interest, the field remains primarily focused on evaluating biological performance, leaving a significant gap in the systematic understanding of nanoparticle fabrication and surface engineering. Current research lacks standardized protocols and rigorous comparisons of extraction and purification methods, which often leads to batch-to-batch variability and inconsistent physicochemical profiles. Furthermore, the complex correlations between synthesis parameters—such as solvent conditions, assembly techniques, and post-synthetic functionalization—and the resulting biological outcomes remain insufficiently explored [1].

To address these challenges, this review provides a systematic overview of the critical stages in SINP development, encompassing extraction, purification, nanoparticle fabrication, and strategic surface modification. This review first delineates the natural biogenesis of melanin and evaluates prevailing methodologies to isolate high-quality squid ink-derived melanin. Subsequently, it synthesizes recent advancements in fabrication techniques and diverse functionalization strategies tailored to amplify their biomedical potential. By establishing key process-property-function correlations, this work serves as a rational design roadmap essential for the clinical translation and industrial scalability of SINP-based therapeutics.

This review aims to provide a comprehensive overview of the fabrication, modification, and biomedical applications of squid ink-derived materials. Various terms, such as cuttlefish ink-derived nanoparticle (CINP), cuttlefish melanin nanoparticle (CMN), and melanin nanoparticle (MNP), appear in the literature depending on the study; however, this review collectively refers to these as SINPs for the sake of clarity.

## 2. Fabrication of Squid Ink-Derived Nanoparticles

### 2.1. Natural Melanin Formation in Squid Ink Subsection

Squid ink is composed of densely packed melanin-bearing granules that originate within the highly specialized epithelial cells lining the ink gland [18]. Across cephalopod species, the pigment is produced inside the ink sac through a tyrosinase-mediated oxidation cascade of catecholamine precursors, a biosynthetic route that closely mirrors the fundamental steps of vertebrate melanogenesis [19,20]. As the ink gland develops, the activity of tyrosinase rises substantially, driving the sequential oxidation of l-tyrosine into l-DOPA, dopamine, and ultimately dopaquinone. This early enzymatic oxidation serves as the critical trigger for the downstream polymerization reactions that culminate in eumelanin production [21]. Inside the lumen of the ink gland, dopaquinone undergoes a series of non-enzymatic redox shifts and intramolecular ring-closure reactions, giving rise to multiple transient catechol species. These intermediates subsequently engage in oxidative coupling and aggregate stepwise, ultimately forming the insoluble eumelanin polymer [22]. The assembled pigment ultimately becomes incorporated into a surrounding network of proteins and mucopolysaccharides, a composite structure that imparts the distinctive density, flow properties, and dispersibility observed in cephalopod ink. Cephalopod ink melanogenesis proceeds through a conserved o-quinone-mediated oxidative polymerization pathway, and the manner in which the pigment is packaged within the ink gland imposes distinct physicochemical characteristics that vary among species.

The classical Raper–Mason model of melanogenesis identifies the tyrosinase-mediated oxidation of dopamine into dopaquinone as the key initiating reaction that drives the downstream sequence of chemical transformations leading to melanin formation [23]. This rate-determining reaction generates a highly unstable quinone intermediate that rapidly converts to dopachrome via intramolecular cyclization. After its formation, dopachrome is converted into the indolic intermediates 5,6-dihydroxyindole (DHI) and 5,6-dihydroxyindole-2-carboxylic acid (DHICA), either through the catalytic activity of dopachrome tautomerase or through spontaneous chemical rearrangements. These monomeric indoles then undergo a series of oxidative steps followed by radical-mediated coupling and structural cross-linking, gradually giving rise to the complex, high-molecular-weight polymer recognized as eumelanin. In this way, melanogenesis progresses from the initial oxidation of catecholamines to dopachrome formation, followed by the generation of indole derivatives and, ultimately, the assembly of the final macromolecular pigment. Squid ink melanin follows this established biochemical route, with the dopamine-to-dopaquinone conversion serving as the central reaction that shapes both the structure and the functional characteristics of the resulting eumelanin pigment.

Beyond its biosynthetic origin, the physicochemical identity of squid ink melanin is defined by its heterogeneous eumelanin structure and hierarchical organization [24,25]. Elemental and spectroscopic studies show that melanin isolated from squid ink contains predominantly carbon and oxygen—typically accounting for roughly half and one-quarter of its composition, respectively—while smaller proportions of nitrogen, sulfur, and various marine-derived inorganic ions, including sodium, magnesium, calcium, and chloride, are also detected [26]. At the supramolecular level, squid ink melanin exhibits a hierarchical organization characteristic of natural eumelanin [27]. Electron microscopy studies showed that squid ink-derived melanin assembles into roughly spherical nanosized particles, typically on the order of 100–200 nm, which can associate into larger submicron or micron-scale aggregates under native conditions [24]. These granules display partially ordered π–π stacking between aromatic sheets with an interplanar spacing of roughly 0.37 nm, consistent with an amorphous, graphite-like arrangement punctuated by heteroatoms. This arrangement allows melanin to integrate the stiffness of its aromatic framework with the variability of its chemical composition, resulting in a pigment that is both mechanically durable and capable of diverse functional behaviors.

In aqueous environments, squid ink melanin demonstrates dynamic colloidal behavior modulated by pH, ionic strength, and the presence of associated proteins and polysaccharides [13,28]. The colloidal stability of melanin nanoparticles is strongly influenced by environmental pH, as shown by studies demonstrating the formation or stabilization of soluble melanin nanoparticles even under acidic conditions when produced enzymatically [29]. Surface carboxyl and phenolic groups contribute to electrostatic stabilization and metal chelation, while the inherent hydrophobicity of the indolic core drives self-association [30]. These physicochemical characteristics give squid ink melanin its notable functionality, such as optical absorption, radical-scavenging capacity, metal-binding ability, and colloidal stability, properties that underpin its increasingly recognized value as a biofunctional marine-derived material [13,28,31].

### 2.2. Extraction of Nanoparticle Formulation from Squid Ink

The extraction of melanin-rich material from squid ink typically begins with isolating intact ink sacs under chilled conditions, a standard practice used to prevent temperature-induced oxidative rearrangements of the pigment [32,33]. The harvested ink is usually mixed with deionized water or a buffered saline solution to lower its viscosity and facilitate even dispersion of the melanin granules, which are originally embedded in a matrix composed of proteins and mucopolysaccharides [19]. The crude ink mixture is first clarified by gentle centrifugation for 10–20 min at 4 °C, which removes coarse particulates such as cellular remnants, insoluble material, and proteinaceous components associated with the pigment, followed by high-g centrifugation to obtain nanosized squid ink particles. This step yields a concentrated pellet of refined melanin granules [34]. In most cases, this washing and centrifugation step is performed at least twice to facilitate the efficient removal of mucus, residual enzymes, and other organic impurities from the ink matrix. During the extraction process, ultrasonic treatment can be applied to increase the dispersibility of squid ink nanoparticles.

The purification of high-purity SINPs involves several rigorous steps, including acidic deproteinization with HCl to dissociate melanin–protein complexes and organic-solvent washing with acetone, ether, or methanol to eliminate lipophilic contaminants and organic impurities [35,36,37]. Such rigorous purification is critical, as residual lipids or proteins can significantly alter the physicochemical profiles and biological interactions of the nanomaterial [38]. Typically, the purified SINPs are washed several times with deionized water and re-suspended in an aqueous solution for immediate use. Although these extraction protocols appear straightforward, parameters such as pH, temperature, and solvent composition exert profound influences on the final yield, aggregation state, and chemical integrity of the SINPs, necessitating strictly controlled and standardized workflows.

### 2.3. Purification and Fabrication Strategies

To refine crude squid-ink melanin into structurally uniform and application-ready nanoparticle formulations, a diverse array of purification and fractionation strategies has been established. Dialysis remains the quintessential method due to its operational simplicity and scalability. By utilizing membranes with a molecular-weight cutoff (MWCO) of 3–10 kDa, dialysis effectively eliminates salts, low-molecular-weight organic residues, and destabilizing metal ions [28]. Extended dialysis (24–72 h with frequent buffer exchange) minimizes ionic strength, thereby fostering the formation of stable, water-soluble melanin fractions [39,40]. However, despite the high purity achieved, the prolonged processing times and substantial water consumption limit its efficiency for high-throughput production.

As more controlled and scalable alternatives, membrane-based separation techniques—encompassing centrifugal ultrafiltration for bench-scale processing and tangential-flow filtration (TFF) for larger volumes—have gained prominence. The application of MWCO membranes (10–100 kDa) facilitates the segregation of low-molecular-weight oligomers from higher-order melanin assemblies. This process yields nanoparticle populations characterized by narrowed polydispersity and enhanced reproducibility [28]. Notably, TFF is particularly advantageous for clinical translation and industrial-scale workflows, as it enables continuous operation while significantly mitigating batch-to-batch variability.

Beyond traditional purification methods, size exclusion chromatography (SEC) can be utilized as a specialized alternative to fractionate melanin oligomers and higher-order aggregates based on their hydrodynamic radii. This strategic approach allows for the isolation of highly defined subpopulations, enabling a more granular analysis of how distinct particle sizes correlate with specific optical and redox characteristics [41].

While squid-ink melanin naturally exists as nanoscale granules, rigorous downstream processing is essential to reproducibly generate homogeneous SINPs tailored for biomedical applications. The transition from crude aggregates to stable colloids typically initiates with mechanical disintegration. Probe sonication, for instance, facilitates the breakdown of these clusters, yielding colloidally stable SINPs with hydrodynamic diameters typically ranging from 80 to 200 nm [28]. To further refine size distributions, mechanical and physicochemical parameters can be precisely modulated. Low-frequency ultrasonication (20–40 kHz) effectively disrupts loosely bound aggregates, while high-pressure homogenization (e.g., microfluidization) ensures superior monodispersity by subjecting suspensions to intense, uniform shear [42].

Finally, strategic surface engineering may be integrated during or after fabrication to enhance functionality. The introduction of stabilizers—such as polyethylene glycol (PEG), polyvinyl alcohol (PVA), or serum proteins—imparts steric hindrance, effectively preventing uncontrolled secondary aggregation. Alternatively, post-synthetic modifications, including oxidative annealing with H_2_O_2_, can be employed to tune the DHI/DHICA ratios [43]. Such molecular-level tuning allows for the precise modulation of antioxidant capacity, metal-binding affinity, and surface charge. Collectively, these integrated strategies ensure the reliable production of SINPs with controlled dimensions and application-specific performance characteristics.

## 3. Functionalization and Modification Strategies of SINPs

The intrinsic physicochemical properties of SINPs—including broadband optical absorption, robust radical-scavenging activity, and high metal-binding capacity—render them exceptionally attractive for diverse biomedical and material applications. However, native SINPs often exhibit significant variability in surface chemistry, hydrophilicity, and colloidal behavior due to their natural origin and the heterogeneous nature of their eumelanin composition [19]. Such inherent diversity can compromise batch-to-batch reproducibility and limit targeted biological interactions, posing challenges for their use in precision drug delivery and advanced imaging.

To overcome these limitations and optimize application-specific performance, functionalization of SINPs is essential. Physicochemical modifications focus on modulating surface charge, dispersibility, and redox states, thereby enhancing stability and tailoring interactions with therapeutic cargos [44]. Complementarily, biofunctional and targeting modifications provide sophisticated control by enabling receptor-specific binding, stimuli-responsiveness, and enhanced cellular trafficking [45]. Collectively, these strategic interventions transform native SINPs into customizable and versatile nanoplatforms with expanded potential across biotechnology, medicine, and environmental engineering.

### 3.1. Physicochemical Modification Strategies

Physicochemical functionalization of SINPs typically begins with surface engineering approaches designed to improve colloidal stability and mitigate aggregation in complex physiological environments. PEGylation is widely favored for its ability to provide robust steric stabilization, which significantly increases systemic circulation time and minimizes non-specific protein adsorption. When applied to melanin nanoparticles, PEGylation imparts superior dispersibility in both aqueous and protein-rich biological media [46]. Alternatively, modifying SINPs with polymers such as polyvinyl alcohol (PVA) can facilitate the construction of defined three-dimensional architectures [47]. Consequently, these polymer-based strategies are instrumental in regulating the delicate balance between the internal hydrophobic interactions of the melanin core and the external hydrophilic repulsion at the nanoparticle interface. By modulating this physicochemical equilibrium, researchers can ensure long-term colloidal stability while tailoring the surface for specific biological environments.

Metal-ion coordination represents a sophisticated physicochemical strategy to alter the optical, electronic, and redox properties of SINPs. The intrinsic catechol and quinone groups of the melanin matrix exhibit a high affinity for transition metals, such as Fe^3+^, Cu^2+^, and Mn^2+^, forming stable chelate complexes [48,49]. These coordination interactions can significantly enhance photothermal conversion efficiency, modify electron-transfer kinetics, and tune radical-scavenging activity [50,51]. Furthermore, such metal binding often induces coordination-driven crosslinking, which can be leveraged to increase the structural compactness and morphological stability of the nanoparticles.

In tandem with metal coordination, redox state modulation offers a means to refine the internal composition of melanin [52]. Controlled oxidation or reduction allows researchers to precisely adjust the DHI/DHICA ratio, thereby tuning the optical, paramagnetic, and antioxidant properties of the melanin.

Further physicochemical modification can be achieved by incorporating hydrophobic moieties, such as alkyl chains or fatty acids, to facilitate the self-assembly of nanosized vesicles; this introduces amphiphilic behavior that enhances compatibility with lipid membranes and improves the encapsulation of hydrophobic drug payloads [53]. Such engineered interfaces expand the utility of SINPs for encapsulation, imaging, and combination therapies. Collectively, these physicochemical modification strategies enable precise control over nanoparticle stability, morphology, electronic characteristics, and functional responsiveness, paving the way for tailored SINP formulations.

### 3.2. Biofunctional/Targeting Modification Strategies

Biofunctional modifications transform SINPs into active nanomaterials, granting them the capacity for receptor-specific targeting and enhanced cellular uptake for precise therapeutic interventions [54]. By incorporating functional peptides such as RGD or iRGD, SINPs can be engineered to engage in high-affinity, receptor-mediated interactions with integrin-overexpressing tumor cells [55]. By specifically docking onto cancer cells, RGD-functionalized SINPs provide a translational pathway to address aggressive and treatment-resistant neoplasms, ensuring both the efficiency and precision required for advanced clinical applications. Carbohydrate-based functionalization, employing moieties such as mannose, galactose, hyaluronic acid (HA), or chitosan, is a widely adopted strategy to transition various nanomaterials into targeted delivery systems [56,57,58]. For instance, the decoration of nanoparticles with mannose promotes specific uptake by dendritic cells and macrophages through mannose receptor-mediated endocytosis, thereby significantly enhancing their potential for immunomodulatory and drug delivery applications [56,59]. Similarly, functionalization with HA enables the selective targeting of CD44-overexpressing cancer cells, leveraging the natural affinity of HA for receptors that are frequently upregulated in various malignant tumors [60,61]. Furthermore, the incorporation of chitosan imparts a positive surface charge and mucoadhesive properties to the nanoparticles, facilitating enhanced penetration across biological barriers and improving the localized delivery of therapeutic payloads [62].

While peptides and polysaccharides provide effective targeting based on broad receptor affinities, antibody or aptamer functionalization offers an even higher degree of precision [63,64,65]. These ligands are capable of distinguishing subtle molecular signatures on target cells, thereby enabling highly selective binding and minimizing off-target interactions in complex biological environments.

Furthermore, hybrid biofunctionalization, achieved by integrating SINPs with functional cargos such as nucleic acids or specific proteins, establishes a synergistic platform where the intrinsic properties of melanin and the targeted bioactivities of the cargo co-exist [66]. These bio-hybrid mediators enable complex biological modulation that transcends the capabilities of single-component systems.

Together, these biofunctional and targeting strategies enable SINPs to engage in receptor-mediated signaling, selective tissue accumulation, and precise immunological interactions. Such modifications effectively transform SINPs from simple natural nanomaterials into highly customizable, multifunctional biomaterials. This evolution highlights the extraordinary versatility of squid ink-derived nanostructures, establishing them as a dynamic platform for diverse biomedical applications that extend to advanced, multi-functional biomaterial strategies.

## 4. Formulation and Hybridization Platforms of Functionalized SINPs for Biomedical Applications

The fabrication and surface functionalization methodologies outlined in Section 2 and Section 3 collectively establish the foundational physicochemical principles for converting crude squid ink-derived melanin into structurally defined nanoscale materials. Nevertheless, the successful translation of squid ink nanoparticles into biomedical applications demands precisely tuned engineering approaches to broaden and refine their functional performance. In biomedical systems, nanoparticles seldom act as isolated agents; rather, they are integrated into higher-order architectures, such as hydrogels, microneedles, hybrid nanoparticles, polymer matrices, or two-dimensional composites to fulfill therapeutic or diagnostic functions [67,68,69,70]. Thus, rational formulation and hybridization play a critical bridging role, integrating the intrinsic molecular properties of functionalized SINPs into nano- to macroarchitectures that govern their biological performance, delivery route, and translational potential.

Furthermore, the chemical structure of melanin enables squid ink-derived nanoparticles to participate in a wide range of intermolecular interactions, including hydrogen bonding, π–π stacking, metal coordination, electrostatic association, and polymer entanglement—facilitating the formation of composite architectures with emergent physicochemical properties that are not achievable by unmodified SINPs alone [71]. These modification approaches can modulate the release profile of drugs, enhance tissue retention, improve mechanical integrity, or introduce multi-modal functionalities such as photothermal activation, sensing capability, or targeted drug delivery [72,73,74]. Consequently, the following sections categorize formulation and hybridization strategies that leverage functionalized SINPs to construct biomedical platforms ranging from polymer hydrogel systems and drug-loaded carriers to detection devices and 2D hybrid materials. These engineered formulations represent the translational backbone through which SINPs evolve from fundamental nanomaterials into clinically and technologically relevant biofunctional platforms.

### 4.1. SINP–Hydrogel and Microneedle Systems

Harnessing functionalized SINPs into polymer-based systems, such as hydrogels or microneedles, has emerged as one of the earliest and most adaptable strategies for translating their inherent physicochemical properties into therapeutically relevant formats. Hydrogels provide a hydrated three-dimensional network capable of accommodating SINPs while preserving their biological activities, and they offer tunable mechanical and diffusional properties that are well-suited for topical, implantable, or wound-healing applications [75,76,77]. Microneedle formulations based on polymeric materials have been developed as specialized topical administration systems to facilitate transdermal delivery of various pharmacological agents [78,79]. By enabling precise, minimally invasive transdermal access, microneedles further enhance delivery efficiency while maintaining patient comfort and compliance [80]. In light of the inherent limitations of unmodified squid ink nanoparticles, their incorporation into polymeric matrices substantially enhances in vivo stability, facilitates sustained release, and improves tissue-specific retention, thereby broadening their therapeutic potential. Following this progress, recent studies have explored a variety of hydrogel and microneedle formulations incorporating SINPs to exploit their functional attributes across therapeutic, regenerative, and diagnostic applications.

**Figure 1:**
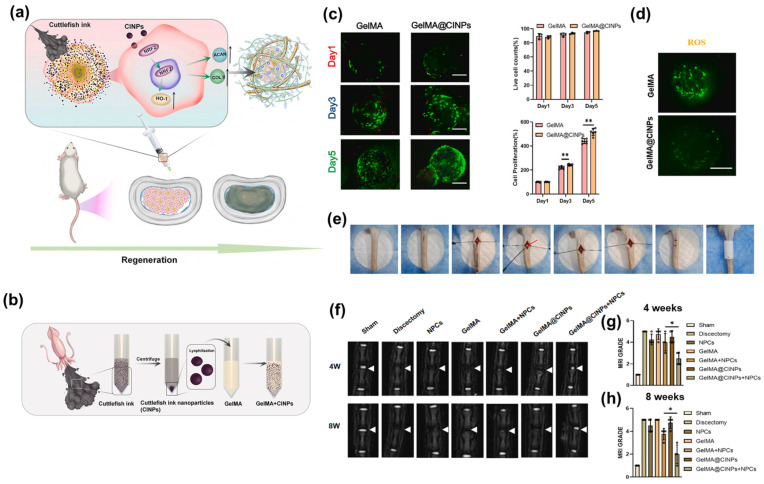
Schematic illustration and experimental validation of SINP-integrated GelMA microspheres for intervertebral disc regeneration through antioxidant activity and NRF2/HO-1 pathway activation. (**a**) Schematic showing the therapeutic mechanism where GelMA@SINPs neutralize oxidative stress to promote nucleus pulposus (NP) cell regeneration and tissue repair. (**b**) Step-by-step fabrication process of SINP-loaded GelMA microspheres from raw cuttlefish ink. (**c**) In vitro Live/Dead staining images and corresponding cell counts/proliferation assays, demonstrating the high biocompatibility and pro-survival effect of SINPs over 5 days. ** (*p* < 0.01) (**d**) Fluorescence images of intracellular ROS scavenging, highlighting the potent antioxidant capacity of SINPs in NPCs under oxidative stress. (**e**) Surgical procedure for the needle-puncture-induced disc degeneration model. (**f**) Representative T2-weighted MRI scans at 4 and 8 weeks post-treatment, showing preserved disc height and hydration in the GelMA@SINPs+NPCs group. (**g**,**h**) Quantitative MRI grading scores at (**g**) 4 weeks and (**h**) 8 weeks, indicating a significant reduction in degeneration severity compared to the discectomy and GelMA-only groups. * (*p* < 0.05) (Note: The labels CINPs in the figure panels refer to SINPs in this study for consistency.) Adapted from [81], under the terms of the Creative Commons Attribution (CC BY) License. Copyright 2025 The authors.

In a recent study, Jin et al. engineered an antioxidant-functionalized scaffold by encapsulating SINPs within a gelatin methacrylate (GelMA) hydrogel matrix [81]. By leveraging a mild extraction protocol, the authors preserved the bioactive profile of the SINPs, comprising eumelanin, amino acids, and monosaccharides, thereby imparting intrinsic radical-scavenging capabilities to the system (Figure 1). This fabrication strategy enabled uniform distribution of SINPs within the hydrogel network while preserving biocompatibility and structural integrity suitable for cell delivery. Beyond material development, the GelMA@SINPs microspheres exhibited significant therapeutic efficacy. These microspheres enhanced nucleus pulposus cell viability and promoted extracellular matrix synthesis by mitigating oxidative stress. This was achieved through a dual-action antioxidant approach, involving physicochemical mechanisms (HAT/SET) and the biological activation of the NRF2/HO-1 signaling pathway, which collectively attenuated intervertebral disc degeneration in vivo. This work demonstrates a robust framework for engineering SINP-incorporated hydrogels, where the photothermal responsiveness and antioxidant capacity of SINPs synergize with the three-dimensional structural support of the scaffold to facilitate advanced tissue regeneration.

Zhao et al. reported a sophisticated SINP–hydrogel platform designed for dual-action therapy in acute radiation syndrome (ARS) and combined radiation–wound injury (CRWI) [82]. In this study, SINP were isolated from fresh cuttlefish ink through sequential ultrasonic disruption, filtration, repeated water washing, and freeze-drying, yielding monodisperse spherical nanoparticles (~134 nm) with characteristic amorphous eumelanin structure and rich surface functional groups (–OH, –COOH, –NH_2_). The authors subsequently integrated SINPs into a sodium alginate (SA) and poly(vinyl alcohol) (PVA), utilizing borax to formulate an injectable composite hydrogel (PS@SINPs) with robust structural integrity. The resulting PS@SINPs hydrogel displayed substantially enhanced mechanical strength, improved crosslinking density, and robust antioxidant activity, attributable to the redox-active melanin core. Under 808 nm NIR irradiation, the hydrogel exhibited strong photothermal conversion, enabling efficient photothermal antibacterial activity against *E. coli* and *S. aureus*. In CRWI mouse models, PS@SINPs significantly accelerated the wound healing process by enhancing angiogenesis through the upregulation of CD31 and VEGF, while simultaneously reducing apoptosis as evidenced by increased BCL-2 expression. Collectively, this study demonstrates the promise of SINP-integrated hydrogels as multifunctional biomaterials that synergize intrinsic antioxidant activity, immunomodulation, and photothermal antibacterial properties to promote tissue repair.

By integrating SINPs and ginsenoside Rc into a GelMA framework, Zhang et al. developed the GelMA@Rc/SINP–hydrogel, which leverages the synergistic antioxidant and anti-inflammatory activities of its components [69]. The system leverages the strong NIR-responsive photothermal properties of purified SINPs (100–200 nm) and the intrinsic pro-angiogenic activity of Rc, providing a multifaceted approach to infection control and tissue repair. A GelMA@Rc/SINPs hydrogel was fabricated through UV-mediated crosslinking, showcasing rapid gelation and an interconnected porous structure equivalent to pristine GelMA. The integration of SINPs conferred potent and stable photothermal properties to the hydrogel without compromising its fundamental biophysical characteristics. The composite exhibited high free radical-scavenging efficiency and NIR-triggered photothermal performance while maintaining superior cytocompatibility toward endothelial cells. Biological evaluation revealed that GelMA@Rc/SINPs hydrogels effectively suppressed oxidative stress and downregulated pro-inflammatory cytokines such as IL-6 and TNF-α. Furthermore, the system bolstered regenerative processes by enhancing cellular migration and tube formation while promoting the expression of angiogenic biomarkers such as VEGF and CD31. In infected wound models, the composite system significantly accelerated wound closure while promoting collagen accumulation and attenuating inflammatory infiltration. Infected wound model animals treated with the composite system exhibited accelerated healing and increased collagen density. These improvements were supported by transcriptomic data showing the stimulation of key angiogenic regulators, including HIF-1α and PI3K-AKT, as well as the downregulation of inflammatory pathways such as NF-κB and IL-17. Overall, this study demonstrates that the integration of SINPs with hydrogels and phytochemicals yields a synergistic photothermal–immunomodulatory platform with significant translational potential for the treatment of chronic and infected wounds.

**Figure 2:**
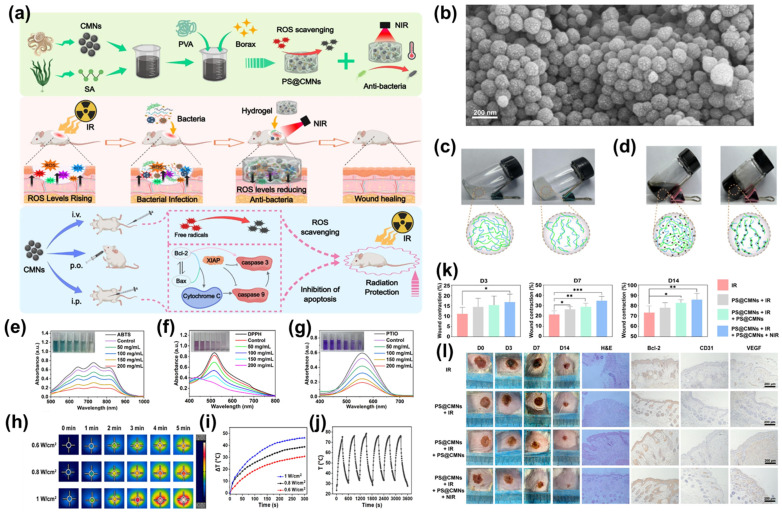
Schematic representation and experimental characterization of PS@SINPs hydrogel for wound healing. (**a**) Preparation and therapeutic regime of PS@SINPs hydrogel. (**b**) Representative SEM image showing the uniform spherical morphology of SINPs (average diameter ~200 nm). (**c**,**d**) Digital photographs and corresponding structural schematics of the (**c**) pristine PS hydrogel and (**d**) SINP-loaded PS@SINPs hydrogel, demonstrating stable integration of the nanoparticles within the polymeric matrix. (**e**–**g**) Concentration-dependent antioxidant assays using (**e**) ABTS, (**f**) DPPH, and (**g**) PTIO radicals, confirming the potent multi-pathway free radical scavenging capacity of SINPs. (**h**–**j**) Photothermal performance of the hydrogel. (**h**) Real-time thermal images, (**i**) power-dependent temperature elevation reaching therapeutic ranges, and (**j**) excellent photothermal stability over five heating/cooling cycles. (**k**,**l**) In vivo wound healing evaluation. * (*p* < 0.05), ** (*p* < 0.01),*** (*p* < 0.001) (**k**) Quantitative wound contraction rates at days 3, 7, and 14, and (**l**) representative photographs of the healing progression alongside histological analysis (H&E and IHC for Bcl-2, CD31, and VEGF) at day 14, highlighting enhanced re-epithelialization and angiogenesis (Note: The labels CMNs in the figure panels refer to SINPs in this study for consistency.) Adapted with permission from [82]. Copyright 2025 Elsevier.

Hao et al. engineered the 3AGM hydrogel as a mechanically reinforced and bioactive wound-dressing system, incorporating natural SINPs and L-arginine-modified polydopamine (APDA) via Ga^3+^ coordination chemistry (Figure 2) [83]. The system focuses on addressing the clinical challenge of multidrug-resistant (MDR) bacterial infections in wound management. Purified through differential centrifugation, the 100–150 nm cuttlefish-derived melanin nanoparticles demonstrated superior broadband NIR-responsive properties, facilitating highly efficient photothermal conversion for therapeutic applications. These SINPs were integrated into an acrylamide/acrylic acid hydrogel network along with L-arginine-assisted polydopamine (APDA) and gallium ions. This multicomponent architecture synergizes photothermal antimicrobial action and antioxidant activity with enhanced mechanical robustness and tissue-adhesive properties. Characterized by its interconnected porosity and enhanced elastic modulus (~564 kPa), the 3AGM hydrogel ensured stable integration with wet biological substrates through strong tissue adhesion. Furthermore, the embedded SINPs enabled high-performance photothermal conversion, elevating the local temperature above 60 °C upon NIR irradiation. The 3AGM platform ensured nearly complete bacterial ablation, exceeding a 99% eradication efficiency against both MRSA and *E. coli* by leveraging its NIR-responsive photothermal properties for a rapid and robust antimicrobial response. The 3AGM platform demonstrated superior biocompatibility and antioxidant capacity, effectively neutralizing ROS and RNS through the dual action of SINPs and APDA. These in vitro findings were complemented by a marked enhancement in fibroblast migration, highlighting the system’s regenerative potential. In vivo evaluation revealed that the hydrogel effectively managed MRSA-infected wounds, evidenced by accelerated closure and minimized inflammatory infiltrates. Key mechanisms included the modulation of IL-6 and TNF-α levels, a strategic shift toward M2 macrophage polarization, and robust CD31-mediated angiogenic activity. Histological analyses demonstrated improved collagen deposition and re-epithelialization. Overall, this study establishes SINP-incorporated hydrogels as a powerful multifunctional platform, bridging the gap between effective photothermal clearance of MDR pathogens and the promotion of endogenous regenerative processes.

To address the complex pathophysiology of vitiligo, Li et al. reported a multifunctional microneedle patch incorporating SINPs, DPG, and skin-derived exosomes [70]. The platform acts as a therapeutic regulator, synergistically neutralizing oxidative stress and suppressing inflammation to restore the cutaneous microenvironment. Purified through sequential high-speed centrifugation, the cuttlefish ink-derived nanoparticles exhibited a spherical morphology with high melanin content. These SINPs were further validated for their strong antioxidant capacity and biocompatibility, making them ideal for integration into the microneedle platform. Due to their melanin-like properties, SINPs exhibited high internalization efficiency in keratinocytes, enabling the effective scavenging of intracellular ROS. This antioxidant action was accompanied by the suppression of vital inflammatory mediators of vitiligo, specifically IL-8, CXCL-16, and HMGB-1. The authors compared three microneedle platforms—fast-dissolving, swellable, and slow-dissolving—to achieve effective transdermal delivery. By offering differentiated drug-release profiles, these systems enabled a customizable approach to addressing the chronic oxidative stress associated with vitiligo. Considering the highly inflammatory and oxidative microenvironment characteristic of vitiligo lesions, a rapid drug-release profile is essential to provide immediate therapeutic intervention. Consequently, the authors prioritized the fast-dissolving (FDMN) and slow-dissolving microneedles (SDMN) for subsequent evaluations to balance both swift action and sustained treatment. The FDMN-mediated delivery of SINPs, DPG, and EXO led to a significant 15.5% improvement in skin repigmentation in vitiligo-induced mice. Notably, this multifunctional platform surpassed the performance of clinical tacrolimus ointment, as evidenced by robust melanin restoration and a visible reduction in skin depigmentation. Mechanistically, the synergistic interplay between its components normalized the pathological microenvironment: SINPs provided antioxidant defense and melanin supplementation, while DPG inhibited HMGB-1 and exosomes promoted the proliferation and migration of melanocytes. Histological analysis further validated these findings, revealing significantly reduced HMGB-1 expression alongside improved melanocyte survival following treatment. This study establishes SINP-loaded microneedles as a powerful multifunctional tool for vitiligo management. By bridging efficient transdermal delivery with multimodal therapeutic action, the platform provides a robust and patient-friendly alternative to conventional dermatological interventions.

### 4.2. SINP–Nanoparticle and Metal-Coordinated Hybrids

While Section 4.1 focused on macro-scale assemblies like hydrogels and microneedles, integrating SINPs with other functional nanoparticles or metal ions at the molecular level offers a different approach to enhancing their intrinsic physicochemical properties. Going beyond polymer-based matrices, these functionalized SINPs readily participate in hybridization with inorganic nanoparticles and transition-metal ions, giving rise to composite architectures with emergent optical, catalytic, and electronic properties. The abundant catechol, quinone, and carboxyl moieties on the melanin surface provide intrinsic coordination sites that strongly bind metal ions, such as Cu^2+^, Fe^3+^, and Mn^2+^, enabling the formation of metal–SINP complexes or metal-bridged nanostructures through chelation and redox-driven assembly. These hybridization processes markedly expand the functional landscape of SINPs by augmenting catalytic antimicrobial activity, modulating oxidative stress for neuroprotection, and accelerating synergistic tissue regeneration. Consequently, a growing body of research has explored diverse SINP–nanoparticle and metal-coordinated hybrid formulations for multifunctional theranostics, sensing, and bioengineering applications.

Lin et al. developed a copper-ion-coordinated nanoplatform by leveraging the metal-chelating capacity of both SINP and synthetic polydopamine (PDA) [84]. This integration resulted in hybrid Cu(II)–melanin and Cu(II)–PDA nanostructures characterized by enhanced antibacterial and wound-healing properties. The naturally derived SINPs, possessing an abundance of catechol, phenolic, and amine functional groups, acted as robust scaffolds for stable Cu^2+^ chelation, forming resilient metal–organic hybrid complexes. These Cu-loaded hybrids exhibited markedly increased ROS-mediated bactericidal activity against *S. aureus* and *E. coli* compared to their metal-free counterparts, while maintaining superior hemocompatibility and cytocompatibility. In vivo evaluations demonstrated that topical application of Cu–melanin hybrids accelerated wound closure and promoted granulation tissue formation by suppressing inflammatory cytokines, notably outperforming pure melanin and PDA nanoparticles. Mechanistic analysis revealed that the sustained, controlled release of Cu^2+^, combined with the intrinsic antioxidant and photothermal properties of melanin, created a synergistic effect that facilitated both bacterial clearance and rapid tissue repair. This study underscores the potential of exploiting SINP’s diverse surface chemistry to engineer therapeutically potent, metal-coordinated hybrids for advanced antimicrobial wound management.

**Figure 3:**
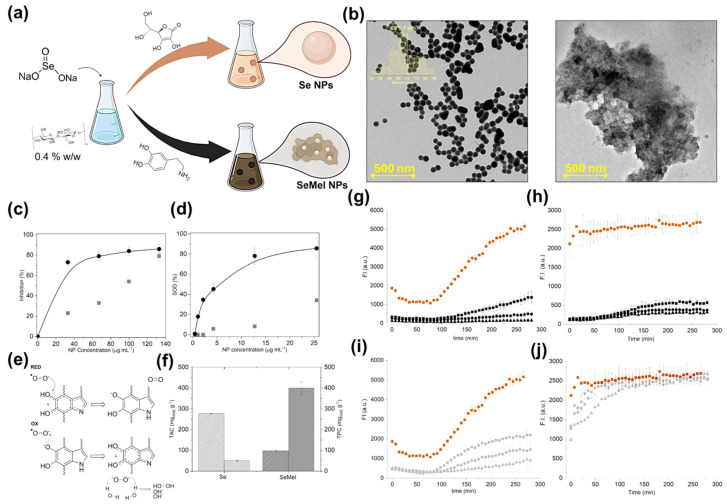
Synthetic strategies and functional evaluations of Se and SeMel NPs. (**a**) Comparative schematic illustration of the green synthesis fabrication process for standard Se NPs and dopamine-modified SeMel NPs. (**b**) Representative TEM images characterizing the morphology: the left panel shows well-dispersed spherical Se NPs, while the right panel reveals the hybrid SeMel NP clusters. Scale bar = 500 nm. (**c**,**d**) Concentration-dependent antioxidant performance showing the superior (**c**) DPPH radical-scavenging inhibition and (**d**) SOD-like catalytic activity of SeMel NPs (black) compared to pristine Se NPs (gray). (**e**) Proposed chemical mechanism for the SOD-like activity, illustrating the synergistic radical neutralization via the melanin-like phenolic structures. (**f**) Quantitative comparison of total phenolic content (TPC) and total ascorbic acid content (TAC), highlighting the significantly higher antioxidant capacity of the SeMel hybrid system. Amyloid inhibition assay: The fluorescence of Aβ21–40 (**g**,**i**) and NPM1264–277 (**h**,**j**) treated with Se NPs (gray) or SeMel (black). Orange symbol represents the NP-free control. Adapted from [85], under the terms of the Creative Commons Attribution (CC BY) License. Copyright 2025 The authors.

To address the complex landscape of neurodegeneration, Pota et al. developed Se@Mel-NPs, a hybrid system that integrates the therapeutic benefits of selenium and melanin (Figure 3) [85]. This platform leverages the synergistic potential of its components to provide a robust defense against amyloid-β fibrillation while maintaining potent antioxidant activity. Selenium nanoparticles were synthesized in situ within a melanin-like polymer matrix, leveraging the redox-active catechol and indole functionalities of melanin to stabilize the Se nuclei and yield uniform hybrid nanostructures. The resulting Se@Mel-NPs exhibited markedly enhanced free-radical-scavenging efficiency, surpassing the performance of each individual component. Furthermore, biochemical assays confirmed that these hybrids effectively suppressed both the fibrillation and oligomerization of amyloid peptides. In addition to their minimal cytotoxicity and superior colloidal dispersion, the Se@Mel-NPs demonstrated significant neuroprotective efficacy. By mitigating oxidative damage in neuronal cell models, these hybrids proved their capacity to preserve cellular viability under stress conditions. The synergistic integration of melanin’s intrinsic redox-buffering capacity with selenium’s catalytic ROS-neutralizing activity underscores the potential of inorganic–organic hybridization. This strategy offers a robust framework for designing therapeutic nanomaterials capable of simultaneously modulating pathological oxidative microenvironments and arresting protein aggregation. Although not SINP-specific, this study reinforces the broader mechanistic foundation for metal-coordinated melanin hybrids, directly supporting the functionalization potential of SINPs in similar therapeutic contexts.

### 4.3. SINP-Based Drug Delivery Platforms

Building upon the various hybrid structures and coordination networks discussed in the previous sections, these engineered SINP systems have been extensively utilized as robust platforms for targeted and controlled drug delivery. Specifically, in parallel with these structural advancements, functionalized SINPs have emerged as versatile carriers that leverage their intrinsic π–π stacking capacity, broad-spectrum hydrophobic interactions, and metal-coordination sites. Coupled with their excellent biocompatibility, these unique physicochemical properties allow for the high-efficiency loading and stimuli-responsive release of diverse therapeutic agents. Unlike many synthetic nanocarriers that necessitate complex surface engineering for guest-molecule encapsulation, SINPs possess naturally heterogeneous aromatic domains and redox-active functional groups that inherently facilitate the loading of small-molecule drugs, polyphenols, photosensitizers, and anti-inflammatory compounds. Their broadband NIR absorption further enables photo-responsive or thermally triggered release profiles, allowing for precise external control over drug liberation within target tissues. Moreover, surface-functionalized SINPs can be engineered for enhanced stability and prolonged circulation, integrating robust drug-loading capability with receptor-specific interactions. Collectively, these attributes position SINPs as an adaptable nanoplatform capable of supporting a diverse range of therapeutic payloads, driving significant interest in SINP-based delivery systems across oncology, dermatology, and regenerative medicine.

Beyond their role as drug carriers, recent findings have highlighted that SINPs can function as stand-alone therapeutic nanoagents. By exerting intrinsic antioxidant, photoprotective, and signaling-modulatory effects independent of additional payloads, SINPs represent a unique class of nanoplatforms that provide therapeutic benefits through their native physicochemical properties. Chen et al. employed an integrative strategy by investigating both synthetic polydopamine nanoparticles (PDA NPs) and natural SINPs as a unified class of bioinspired melanin-like agents [86]. This study evaluated their collective potential as skin-protective barriers to counteract the hyperpigmentation induced by blue light exposure. By positioning both synthetic and natural melanin variants as complementary tools, the researchers demonstrated a versatile approach to dermatological protection. The purification of SINPs involved repeated washing and proteolytic digestion to ensure the removal of protein contaminants. The resulting nanoparticles retained the characteristic aromatic core of melanin, featuring diverse surface functional groups—including amino, hydroxyl, and carboxyl moieties—that facilitate their interaction with biological systems. Synthesized via dopamine self-polymerization, the PDA NPs shared key physical traits with SINPs, including a spherical diameter of 100–200 nm and robust ζ-potential. Notably, both systems demonstrated broad-spectrum absorption profiles analogous to endogenous melanin, ensuring their functional relevance in dermatological applications. Notably, the strong photostability and high blue light extinction coefficients of both PDA NPs and SINPs underpinned their antioxidant efficacy, allowing for the rapid scavenging of ROS produced upon light exposure. Consequently, both PDA NPs and SINPs effectively mitigated the hallmarks of blue light-induced damage, including epidermal hyperplasia and hyperpigmentation, by suppressing tyrosinase-mediated melanogenesis in vitro and in vivo. Mechanistic analysis revealed that these nanoparticles interrupted the FZD2–TYR–melanin signaling axis, a key regulatory pathway in melanogenesis, leading to a significant reduction in pigment formation. These findings underscore the feasibility of SINPs as a bioactive, natural melanin-based nanomaterial. Beyond their inherent properties, this study highlights their significant potential in dermatological applications, particularly for the development of next-generation sunscreens designed to combat blue light-induced pigmentation.

**Figure 4:**
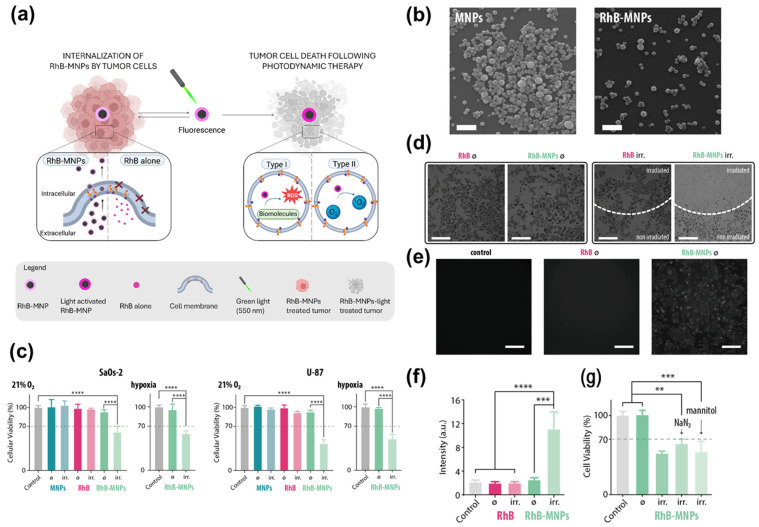
Evaluation of the phototherapeutic potential and intracellular action mechanism of Rhodamine B-conjugated SINPs (RhB-SINPs). (**a**) Schematic illustration showing the endocytosis-mediated internalization of RhB-SINPs into tumor cells and the subsequent induction of cell death via Type I and Type II reactive oxygen species (ROS) pathways following green light (550 nm) irradiation. (**b**) Representative TEM images comparing the morphology of SINPs and RhB-conjugated SINPs, demonstrating that surface functionalization maintains the uniform spherical structure. Scale bar = 1 μm. (**c**) Viability of SaOs-2 and U-87 cells under normoxic and hypoxic conditions, with (irr.) or without (ø) light irradiation. **** (*p* < 0.0001) (**d**) Micrographs of MTT-stained cells treated with RhB or RhB-SINPs under normoxia, with (irr.) or without (ø) light irradiation. Scale bar = 400 μm. (**e**) Fluorescence micrographs of cells treated with RhB or RhB-SINPs for 24 h. Scale bar = 200 μm. (**f**) Intracellular ROS levels in RhB and RhB-SINP-treated cells (24 h) measured by DCFH-DA fluorescence (±irradiation). *** (*p* < 0.001), **** (*p* < 0.0001). (**g**) Investigation of specific ROS mechanisms using inhibitors (NaN_3_ for Type II; mannitol for Type I), verifying that RhB-SINPs utilize both pathways to overcome oxygen-limited tumor microenvironments (Note: The labels MNPs in the figure panels refer to SINPs in this study for consistency). ** (*p* < 0.01), *** (*p* < 0.001). Reproduced with permission from [87]. Copyright 2025 American Chemical Society.

Beyond their stand-alone bioactivity, SINPs serve as efficient delivery vehicles for enhancing the efficacy of conventional therapeutic agents through targeted functionalization. For instance, Caldas et al. developed a specialized photodynamic therapy (PDT) nanoplatform by utilizing SINP derived from Sepia officinalis (the common cuttlefish) (Figure 4) [87]. By loading Rhodamine B (RhB) onto these natural carriers, the researchers addressed the inherent shortcomings of conventional photosensitizers, such as poor aqueous solubility, suboptimal cellular uptake, and oxygen-dependent therapeutic efficacy. Purified via mechanical homogenization and sonication, the natural SINPs displayed a spherical diameter of 180–200 nm. Notably, these nanoparticles exhibited excellent colloidal stability, facilitating their use as reliable delivery vehicles. RhB was integrated on the melanin surface with a remarkably high loading efficiency (94.3%) via noncovalent interactions, including π–π stacking and hydrogen bonding. The SINP-based delivery system facilitated a dramatic increase in RhB solubility and cellular uptake. Confocal microscopy confirmed robust internalization and subsequent perinuclear trafficking, establishing the necessary spatial foundation for potent photodynamic activity upon irradiation. Therapeutically, RhB-SINPs maintained robust PDT activity under hypoxia, overcoming the oxygen-dependent limitations that often render classical Type II photosensitizers ineffective in solid tumors. At a concentration of merely 10 μg/mL, the nanoconstructs induced efficient ROS-mediated apoptosis upon 550 nm irradiation. This high efficacy under low-dose and low-fluence conditions underscores the superior performance of SINPs as a synergistic PDT nanomedicine. Mechanistically, by engaging concurrent Type I and Type II pathways, RhB-SINPs bypassed the oxygen constraints of traditional PDT. Coupled with their favorable biocompatibility, green-chemistry origin, and high photostability, this work establishes melanin nanoparticles as a promising multifunctional platform for oxygen-independent phototherapy.

In conclusion, SINPs are evolving from passive carriers into active, multifunctional therapeutic platforms. By combining inherent bioactivity (antioxidant and photoprotective) with exceptional loading capacity via noncovalent interactions, they address the critical limitations of conventional drugs, such as poor solubility and oxygen-dependent efficacy. Their green-chemistry origin and biocompatibility further validate SINPs as a sustainable and superior alternative for next-generation precision medicine.

### 4.4. SINP-Based Sensing and Detection Systems

In addition to their role as therapeutic carriers, the unique optical and electrochemical characteristics of SINPs—often amplified through the hybridization strategies mentioned in previous sections—enable their application in highly sensitive sensing and detection systems. Specifically, functionalized SINPs possess a unique constellation of redox-active and metal-binding properties, which, when combined with their inherent optical signals, make them exceptionally well-suited for diverse diagnostic and environmental monitoring applications. Their broad-spectrum and stable optical absorption across the visible-to-NIR range facilitates photoacoustic, colorimetric, and photothermal readouts. Simultaneously, the catechol/quinone functional groups provide a strong affinity toward transition metals and reactive oxygen species (ROS), enabling selective detection through absorbance shifts, fluorescence quenching, or electrochemical signaling.

Moreover, strategic surface functionalization offers the potential to further tailor the specificity of SINP-based sensors, theoretically allowing for precise interactions with a broad spectrum of targets, including environmental toxins, metabolic markers, or pathogenic organisms. When integrated into advanced composite systems—such as conductive polymer films, electrode coatings, or hydrogel matrices—SINPs could function as versatile transduction elements, potentially converting diverse chemical or biological stimuli into quantifiable optical or electrical outputs. While recent efforts have focused on clinical biomarkers, these multifaceted attributes suggest that SINP-enabled platforms could be readily adapted for environmental monitoring, food safety analysis, and real-time metabolic tracking.

**Figure 5:**
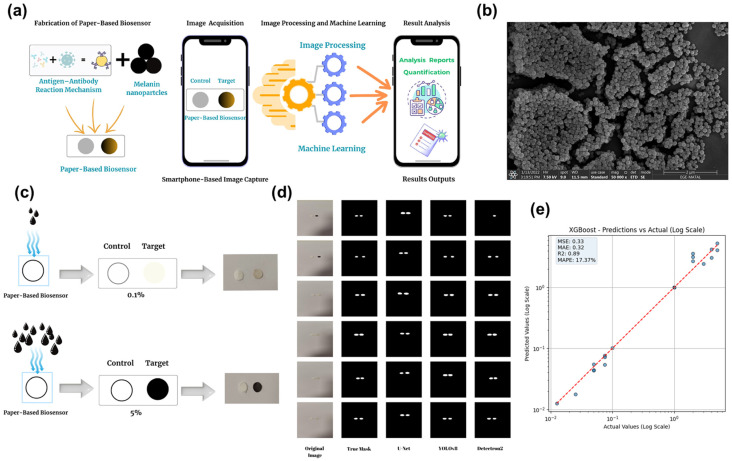
A smartphone-integrated paper biosensor platform utilizing SINPs for colorimetric detection and machine learning-based quantification of biomarkers. (**a**) Schematic overview of the diagnostic workflow, from the fabrication of the SINP-functionalized paper biosensor to smartphone-based image capture and cloud-based machine learning analysis. (**b**) SEM image showing the highly uniform and spherical SINPs, which provide the consistent colorimetric signals necessary for accurate sensing. (**c**) Visual representation of the colorimetric response, where the SINP-laden target region exhibits distinct intensity changes corresponding to biomarker concentrations (0.1% vs. 5%). (**d**) Comparison of original biosensor images and ground truth masks against segmentation predictions from U-Net, YOLOv8, and Detectron2, showing visually comparable performance with minor variations in target region detection. (**e**) XGBoost model performance for biomarker quantification. (Note: The labels MNPs in the figure panels refer to SINPs in this study for consistency.) Adapted from [88], under the terms of the Creative Commons Attribution (CC BY) License. Copyright 2025 The authors.

Wang et al. engineered a portable, AI-integrated biosensing platform by employing SINPs as a sustainable, eco-friendly chromogenic probe for the rapid and cost-effective detection of the cancer biomarker CA19-9 (Figure 5) [88]. Purified through optimized centrifugation and washing cycles, the resulting SINPs exhibited excellent colloidal stability. Integrated into a nitrocellulose-based paper-based analytical device (PAD), the SINPs acted as visual reporters. Antigen-specific binding to immobilized antibodies regulated the localization of the nanoparticles, producing a quantifiable colorimetric response proportional to the presence of the CA19-9 biomarker. The SINP-driven colorimetric changes provided a clear, dose-dependent readout compatible with visual or smartphone-assisted analysis. This reagent-free approach significantly reduces the complexity and cost associated with traditional enzymatic biosensors. For improved field reliability, the platform utilized a comprehensive deep-learning suite. The implementation of U-Net (segmentation), YOLOv8 (detection), and Detectron2 (ROI classification) allowed for the automated and high-fidelity interpretation of the SINP-generated color patterns. This AI-assisted architecture facilitated the robust and automated interpretation of PAD colorimetric patterns, effectively mitigating inconsistencies caused by fluctuating ambient lighting or diverse user-handling conditions. The resulting diagnostic system exhibited exceptional sensitivity and high linearity across clinically relevant CA19-9 concentration ranges, complemented by negligible cross-reactivity and superior stability over repeated measurements. Collectively, this study underscores the potential of SINPs as a high-performance natural alternative to traditional metallic or synthetic nanoparticle probes in paper-based biosensing. By integrating intrinsic melanin bioactivity with deep-learning precision, this platform offers a biocompatible, cost-effective, and highly scalable solution for next-generation point-of-care cancer diagnostics.

### 4.5. SINP–2D Material Hybrid Systems

Expanding from the zero-dimensional and three-dimensional hybrids discussed previously, the integration of SINPs with two-dimensional (2D) materials represents a recent frontier in creating synergistic composites for advanced biomedical engineering. Beyond their role in polymeric matrices or inorganic nanoparticle hybrids, functionalized SINPs have emerged as versatile interfacial modifiers for layered nanostructures, including MXenes and graphene [89]. This integration leverages the high surface area of 2D scaffolds alongside the unique bio-functional properties of SINPs to achieve superior electronic, mechanical, and therapeutic performance. The abundant surface catechol, quinone, and carboxyl moieties enable robust electrostatic interactions, hydrogen bonding, and π–π stacking with 2D sheets. Incorporating SINPs into 2D lattices serves several critical functions: it mitigates the rapid oxidation of MXenes, enhances film uniformity, improves mechanical flexibility, and introduces novel optical or electronic functionalities. Furthermore, SINP-derived coatings have demonstrated potential as biocompatible, conductive, and environmentally stable alternatives to traditional synthetic films in sensing, energy storage, and wearable electronics. These hybrid systems illustrate how the molecular versatility of SINPs can be harnessed to engineer multifunctional 2D composites with synergistic performance characteristics, driving significant interest in SINP–2D hybrid nanoplatforms for next-generation material science.

Liu et al. strategically engineered an intercalated MXene–carbon hybrid structure by incorporating cuttlefish ink-derived carbon nanospheres (CCNS) into Ti_3_C_2_T_x_ MXene layers [68]. This architecture was designed to overcome the intrinsic challenges of pristine MXene, such as irreversible restacking and limited ion accessibility. The CCNS were synthesized by carbonizing purified cuttlefish ink, resulting in uniformly sized, heteroatom-doped nanospheres. To facilitate robust assembly, these nanospheres were functionalized with cetyltrimethylammonium bromide, promoting electrostatic interactions with the negatively charged MXene sheets. The resulting MXene@CCNS composite exhibited significantly expanded interlayer spacing and an enlarged electrochemically accessible surface area by effectively suppressing lamellar collapse. This structural modification enabled more efficient ion diffusion and rapid charge transfer kinetics. These findings highlight the potential of marine-derived carbon nanostructures as effective spacers and stabilizing agents for 2D materials, offering a sustainable and scalable strategy to enhance the structural integrity and energy-storage performance of MXene-based electrochemical devices.

While these advancements primarily demonstrate enhanced energy-storage capabilities, the high conductivity and structural resilience of SINP–MXene hybrids suggest significant potential for biomedical integration. Such multifunctional composites could be readily adapted as high-performance interfaces for wearable biosensors, neural electrodes, or electronic skins, where the biocompatibility of melanin-derived spacers ensures long-term stability within biological environments. Thus, the synergy between 2D nanomaterials and SINP-based carbon architectures extends the utility of squid ink-derived materials beyond simple fillers, positioning them as foundational components for next-generation multifunctional biomaterials.

## 5. Discussion

### 5.1. Intrinsic Advantages of Natural Squid Ink Melanin

Beyond their versatile applications, the unique biomaterial advantages of SINPs provide compelling reasons to categorize them as a distinctive class of naturally optimized nanomaterials, rather than merely biological analogs of synthetic melanin. SINPs possess intrinsic biocompatibility, broad-spectrum antioxidant activity, immunomodulatory effects, and exceptional photothermal conversion efficiency, all of which arise directly from their native indolic architecture and metal-coordination motifs. Unlike synthetic melanin analogs, which often require complex polymerization, dopants, or post-synthetic modifications to achieve comparable optical or redox properties, SINPs exhibit a naturally broadened absorbance range across the UV–NIR spectrum, stable radical-scavenging capacity, and high-affinity chelation toward biologically relevant metal ions. Furthermore, their inherent nanogranular morphology bypasses the need for artificial nanoparticle fabrication, thereby enabling more predictable biological interactions and mitigating the risks of unintended chemical by-products often associated with synthetic processes. Collectively, these attributes underscore the potential of SINPs to serve as inherently functional and bio-harmonious building blocks for advanced therapeutic and diagnostic technologies, effectively overcoming the limitations typically associated with synthetic melanin-based biomaterials.

### 5.2. Current Research Challenges and Limitations

Despite rapid progress in the extraction, functionalization, and formulation of SINPs, formidable scientific and translational challenges remain before SINP-based systems can be fully realized in clinical practice. The intrinsic heterogeneity of natural melanin, characterized by variable DHI/DHICA ratios, trace metal content, and heterogeneous amino acid compositions, continues to hinder batch-to-batch reproducibility. Such structural inconsistency complicates efforts to establish standardized physicochemical and biological profiles for SINPs. Moreover, current extraction and purification workflows, despite being increasingly refined, often rely on rigorous conditions that can inadvertently alter the native melanin architecture or introduce variability across species, harvesting seasons, and processing environments.

To address these challenges, a transition toward standardized raw material sourcing is essential. Currently, the inherent heterogeneity of SINPs is largely driven by the diverse environmental and dietary conditions of wild-harvested squids. The implementation of controlled squid aquaculture systems could provide a viable solution by ensuring consistent physiological conditions, thereby minimizing batch-to-batch variability in melanin composition. By establishing standardized breeding and harvesting protocols, a more predictable chemical profile of SINPs can be achieved. This upstream standardization, integrated with advanced analytical techniques such as high-resolution mass spectrometry and machine learning-assisted fingerprinting, will provide the rigorous quality control and chemical identity required for regulatory approval and large-scale clinical translation. Beyond the fundamental issues of standardization, the successful clinical integration of SINPs necessitates addressing specific technical hurdles unique to each biomedical domain. The current limitations and strategic research directions for diverse SINP-based applications are summarized in Table 1.

### 5.3. Future Research and Translational Direction

Looking ahead, the development of next-generation functionalization strategies offers a promising avenue for enhancing the precision, tunability, and therapeutic versatility of SINPs. The integration of advanced chemical strategies, such as bioorthogonal conjugation, enzyme-directed modification, and metal–phenolic coordination networks, could facilitate more predictable control over surface properties and enable hierarchical self-assembly into programmable nanostructures. Meanwhile, emerging formulation platforms, including hybrid hydrogel-microneedle systems, metal-coordinated nanoclusters, and SINP-enabled 2D composites, highlight the potential for integrating these materials into multifunctional devices capable of simultaneous sensing, imaging, and therapy. However, translating these sophisticated materials into clinical applications will require systematic evaluation of long-term biocompatibility, biodegradation pathways, and immunomodulatory effects, alongside the development of scalable manufacturing practices that comply with stringent regulatory standards.

Establishing reproducible and sustainable natural source management will be paramount to ensuring consistent material properties and achieving the reliability required for medical-grade production. Given that SINPs are derived from natural sources, proving the consistency of their chemical composition is a prerequisite for clinical transition. Future development must transition toward manufacturing processes that comply with GMP standards to ensure that each batch meets rigorous purity and safety specifications. Such regulatory alignment is essential for obtaining approval from agencies like the FDA, ensuring that the natural variability of the raw squid ink is strictly controlled within clinical standards.

## 6. Conclusions

Ultimately, the unique optical, redox, and coordination properties of SINPs establish them as a compelling class of marine-derived functional nanomaterials, offering transformative potential across oncology, regenerative medicine, theranostics, and biosensing. Future progress will likely be driven by interdisciplinary collaboration across marine biochemistry, polymer science, nanotechnology, and biomedical engineering to establish SINPs as reliable, modular platforms for next-generation therapeutic and diagnostic technologies. As foundational barriers in standardization, mechanistic understanding, and translational scalability are progressively addressed, functionalized SINP systems are poised to evolve from experimental materials into clinically relevant nanoplatforms that offer distinctive advantages over their synthetic counterparts.

## Figures and Tables

**Table 1:** Summary of current limitations and future research directions for SINP-based biomedical applications.

Application Field	Current Limitations	Proposed Future Directions
Drug Delivery Systems	Low targetability to disease region, concerns regarding long-term accumulation in the liver/spleen	Fine-tuned engineering of target-specific surface modifications (e.g., ligands, aptamers), systematic biodistribution and clearance kinetics studies
Bio-imaging and Theranostics	Inherent low quantum yield and lack of simultaneous theranostic applications	Developing hybrid nanostructures (e.g., SINP-gold or SINP-carbon dots) to enhance fluorescence and multimodal imaging contrast.
Tissue Engineering and Wound Healing	Batch-to-batch variability in mechanical properties of SINP-bearing scaffolds, unclear degradation rates of SINPs in various tissue types.	Establishing source-to-product standardization (e.g., aquaculture), long-term in vivo studies to monitor degradation behavior
Photothermal Therapy	Relatively low photothermal conversion efficiency in specific NIR windows, potential thermal damage to surrounding healthy tissues	Developing thermally responsive gatekeepers or smart delivery systems for precise, localized heat release

## Data Availability

Data sharing not applicable to this article as no datasets were generated or analyzed during the current study.
